# Group B Streptococcal Membrane Vesicles Induce Proinflammatory Cytokine Production and Are Sensed in an NLRP3 Inflammasome-Dependent Mechanism in a Human Macrophage-like Cell Line

**DOI:** 10.1021/acsinfecdis.4c00641

**Published:** 2025-01-06

**Authors:** Cole R. McCutcheon, Jennifer A. Gaddy, David M. Aronoff, Shannon D. Manning, Margaret G. Petroff

**Affiliations:** † Department of Microbiology, Genetics, and Immunology, Michigan State University, East Lansing, Michigan 48824, United States; ‡ Department of Medicine, Division of Infectious Disease, Vanderbilt University Medical Center, Nashville, Tennessee 37232, United States; § Department of Pathology, Microbiology, and Immunology, Vanderbilt University Medical Center, Nashville, Tennessee 37232, United States; ∥ Tennessee Valley Healthcare System, Department of Veterans Affairs, Nashville, Tennessee 37212, United States; ⊥ Department of Medicine, Indiana University School of Medicine, Indianapolis, Indiana 46202, United States; # Department of Pathobiology and Diagnostic Investigation, Michigan State University, East Lansing, Michigan 48824, United States

**Keywords:** *Streptococcus*, cytokines, inflammation, vesicles, inflammasome, macrophage

## Abstract

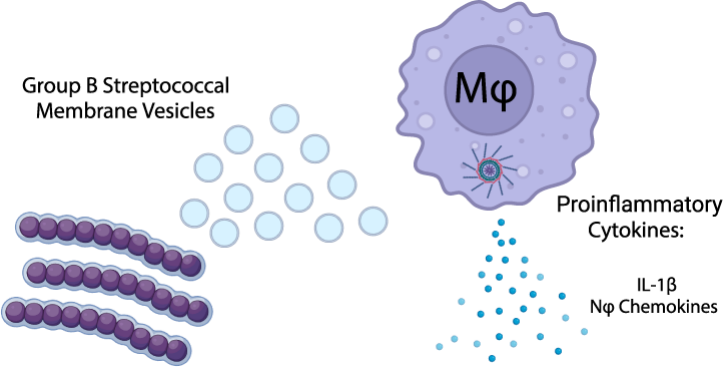

Group B *Streptococcus* (GBS) is a major cause of fetal and neonatal mortality worldwide. Many of the adverse effects of invasive GBS are associated with inflammation; therefore, understanding bacterial factors that promote inflammation is of critical importance. Membrane vesicles (MVs), which are produced by many bacteria, may modulate host inflammatory responses. While it is known that mice injected intra-amniotically with GBS MVs exhibit large-scale leukocyte infiltration, preterm birth, and subsequent fetal death, the immune effectors driving this response remain unclear. Here, we hypothesized that THP-1 macrophage-like cells respond to GBS-derived MVs by producing proinflammatory cytokines and are recognized through one or more pattern recognition receptors. We show that THP-1s produce high levels of neutrophil- and monocyte-specific chemokines in response to MVs derived from different clinical isolates of GBS. Using antibody microarrays and multiplex Luminex assays, we found that GBS MVs elicit significantly (*p* < 0.05) higher levels of CCL1, CCL2, CCL20, CXCL1, CXCL10, and IL-1β relative to untreated THP-1s. Using chemical inhibitors in combination with caspase-1 activity assays and Luminex assays, we further demonstrate that GBS MVs upregulated IL-1β production in a caspase-1 and NLRP3-dependent manner, ultimately identifying NLRP3 as a sensor of GBS MVs. These data indicate that MVs contain one or more pathogen-associated molecular patterns that can be sensed by the immune system and show that the NLRP3 inflammasome is a novel sensor of GBS MVs. Our data additionally indicate that MVs may serve as immune effectors that can be targeted for immunotherapeutics.

Group B *Streptococcus* (GBS) is an opportunistic pathogen that colonizes the vaginal or rectal tract of ∼30% of women.^1^ While maternal colonization is often asymptomatic, GBS can cause severe infections in pregnant women and neonates.^1^ Pregnancy- and neonatal-associated GBS infections are often characterized by pathologies exhibiting a high degree of inflammation. During pregnancy, this can present as placental villitis and preterm birth, whereas in neonates, GBS can cause meningitis and sepsis.^2−4^ Despite the high colonization frequencies in mothers, only a fraction of women and their neonates develop these threatening infections. The reasons for this discrepancy, however, are incompletely characterized.

We and others have postulated that strain variation contributes to the discrepancy in the disease outcome. Indeed, specific phylogenetic lineages of GBS, which are defined by multilocus sequence typing (MLST), are more likely to cause neonatal infections.^5−7^ Notably, sequence type (ST)-17 strains are more commonly associated with invasive neonatal infections,^5,8,9^ whereas ST-1 strains are associated with invasive disease in adults.^10^ Conversely, ST-12 strains have been linked to asymptomatic maternal colonization.^11^ We demonstrated that ST-17 strains elicit stronger proinflammatory immune responses and persist longer inside macrophages than other strains.^12,13^ Interestingly, we also found that ST-1 and ST-17 strains induce stronger activation of the proinflammatory transcription factor NF-κB compared to ST-12 strains.^13^ While ST-17 strains were previously found to have unique virulence gene profiles relative to other lineages, the specific bacterial factor(s) promoting these altered inflammatory responses are not fully understood.^14−16^

Recently, it has been reported that GBS produces membrane vesicles (MVs) that can induce substantial recruitment of neutrophils and lymphocytes into murine extraplacental membranes, which mimicked GBS-associated chorioamnionitis in humans.^17,18^ In support of this finding, GBS MVs induce production of the neutrophil chemokine CXCL1 in a murine model of *in utero* infection,^17,19^ similar to other GBS infection models.^20,21^ Further, we have recently reported that GBS MV production varies in abundance and protein composition across STs.^17,19,22^ More specifically, several immunomodulatory virulence factors, including hyaluronidase, C5a peptidase, and sialidase, were found in MVs; however, these virulence factors were differentially abundant across STs.^22^ Together these data indicate that MVs promote proinflammatory immune responses; however, no prior studies have comprehensively examined the mechanisms by which human leukocytes respond to GBS MVs.

As sentinel leukocytes at the maternal–fetal interface, macrophages play an important role in shaping immune responses. At the maternal–fetal interface, macrophages make up 20–30% of leukocytes^23^ and play pivotal roles in fertility,^24^ placental function,^25^ and host–pathogen interactions.^26−28^ The THP-1 monocytic leukemia cell line can be differentiated with phorbol esters into macrophage-like cells^29^ and serve as a model system to evaluate host responses to GBS.^12,30^ Using this model, we have previously shown that THP-1 cells produce high levels of proinflammatory cytokines in response to GBS. Interestingly, several cytokines displayed lineage-specific inflammatory responses, with ST-17 strains eliciting a more potent inflammatory response compared with other lineages.^13^ Here, we examined macrophage responses to GBS MVs isolated from a diverse set of strains and found that these MVs induce the production of proinflammatory cytokines and chemokines. We also identified NLRP3 (nucleotide-binding oligomerization domain, leucine-rich repeat and pyrin domain-containing receptor-3) as a sensor of GBS-derived MVs. In summary, this study has expanded our current understanding of how host cells respond to GBS MVs. Additionally, by interrogating the mechanism of MV action, we identified the proinflammatory pathways and receptors that could be used as potential immunotherapeutic targets.

## Results

### GBS MVs Elicit Proinflammatory Cytokine Responses

**Figure 1:**
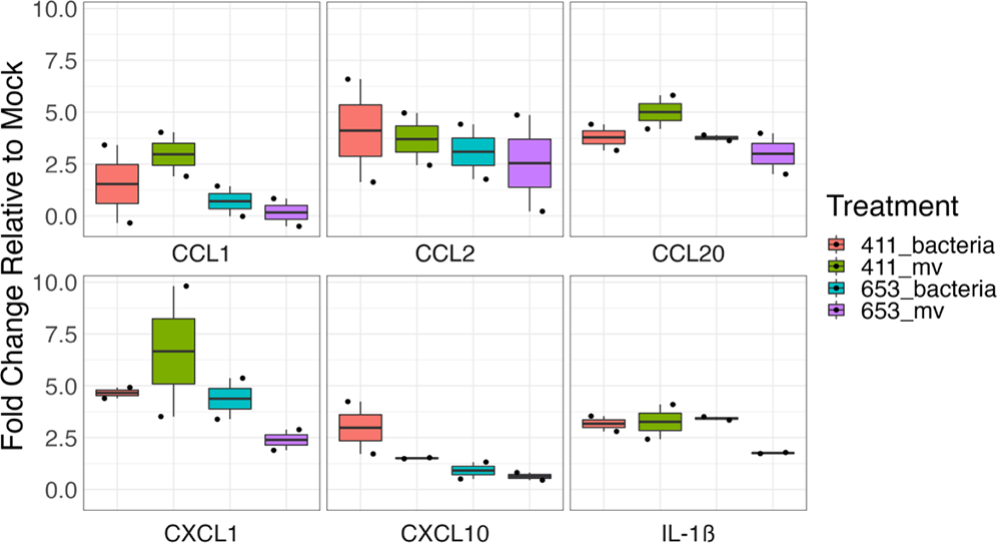
Human antibody cytokine microarray reveals highly upregulated cytokines in response to GBS and GBS MVs. Human cytokine antibody microarrays (Abcam) were probed with supernatants from untreated, bacteria-treated, or MV-treated THP-1-derived macrophages. Membrane densitometry was assessed using ImageJ software. The bacterial strains used are an invasive ST-17 strain (GB411) and a colonizing ST-12 strain (GB653). Shown here are hits of interest that displayed greater than 2-fold change (FC) induction relative to untreated controls in at least one group. Black dots indicate a single biological replicate. *n* = 2/treatment.

We first sought to characterize the cytokine responses elicited by MVs from a diverse set of GBS strains representing some of the most common STs in circulation. Initially, human cytokine antibody microarrays were performed to examine responses for the invasive ST-17 (GB411) strain and colonizing ST-12 (GB653) along with their cognate MVs. This analysis revealed that MVs induced cytokine production from THP-1-derived macrophage-like cells. Of the 80 cytokines and chemokines assayed, seven were upregulated at least 2-fold relative to the untreated cells (Figure S1). Specifically, MVs upregulated the monocyte and neutrophil chemokines, CCL1, CCL2, CXCL1, and CCL20, the pyrogen IL-1β, and the proinflammatory cytokine IL-6 (Figures 1 and S1). Several cytokines were also differentially induced, as MVs from GB411 induced CXCL1, CCL1, and IL-1β more strongly than MVs from GB653 (Figure 1). However, similar responses were not elicited from the THP-1 cells when only bacterial cells were examined, and no difference was detected between GB411 and GB653 (Figure 1).

**Figure 2:**
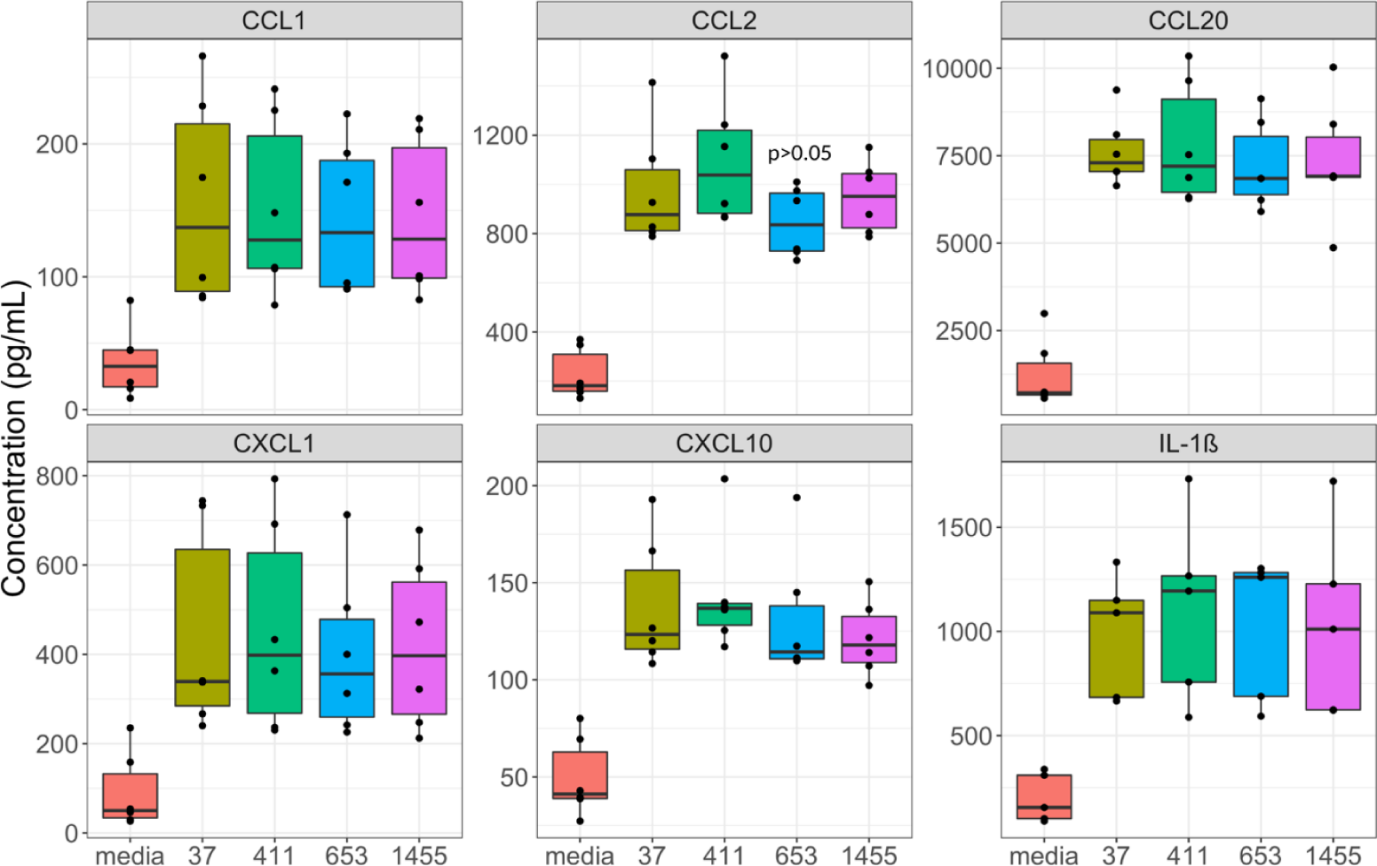
MVs induce proinflammatory cytokine and chemokine responses. Supernatants from THP-1-derived macrophages which were untreated or treated with MVs (MOI 100) for 25 h were assessed for cytokine production using ProcartaPlex multiplex or singleplex (IL-1B) bead-based assays. The MVs were derived from the indicated bacterial strains, an invasive ST-17 strain (411), a colonizing ST-12 strain (653), an invasive ST-1 strain (37), or an invasive ST-12 strain (1455). Individual black dots indicate a single biological replicate (*n* = 5–6 for each group). Statistics were determined using either an ANOVA with a Tukey HSD post hoc or a Kruskal–Wallis test with a Dunn Test post hoc when appropriate. All comparisons to mock treatment were significant (*p* < 0.05) unless noted with a specific p-value.

To validate the differences in cytokine production, quantitative Luminex-based assays were used. Consistent with previous results,^13^ GBS induced a potent proinflammatory response relative to untreated controls, though IL-6 production remained unchanged by MV exposure (Figures S2 and S3). Moreover, the MVs induced CCL1, CCL20, CXCL10, CXCL1, and IL-1β, but no differences were observed between the strains from which the MVs were derived (Figure 2). Although CCL2 displayed an elevated response relative to mock treatment, this induction was significant only for MVs produced by the three invasive strains (GB37, GB411, and GB1455).

To ensure that these cytokine responses were not biased due to differential levels of cell death, we evaluated the cytotoxicity for all strains using a lactate dehydrogenase activity assay. Notably, the bacteria induced moderate levels of cytotoxicity that varied slightly across strains, with the nonhemolytic strain, GB37, inducing more cytotoxicity than GB1455 (Figure S4). Although low levels of cytotoxicity (average ∼6%) were observed during MV treatments, no difference in cytotoxicity levels was observed among MVs produced by the four different GBS strains.

### GBS MVs Induce Caspase-1 Activation

**Figure 3:**
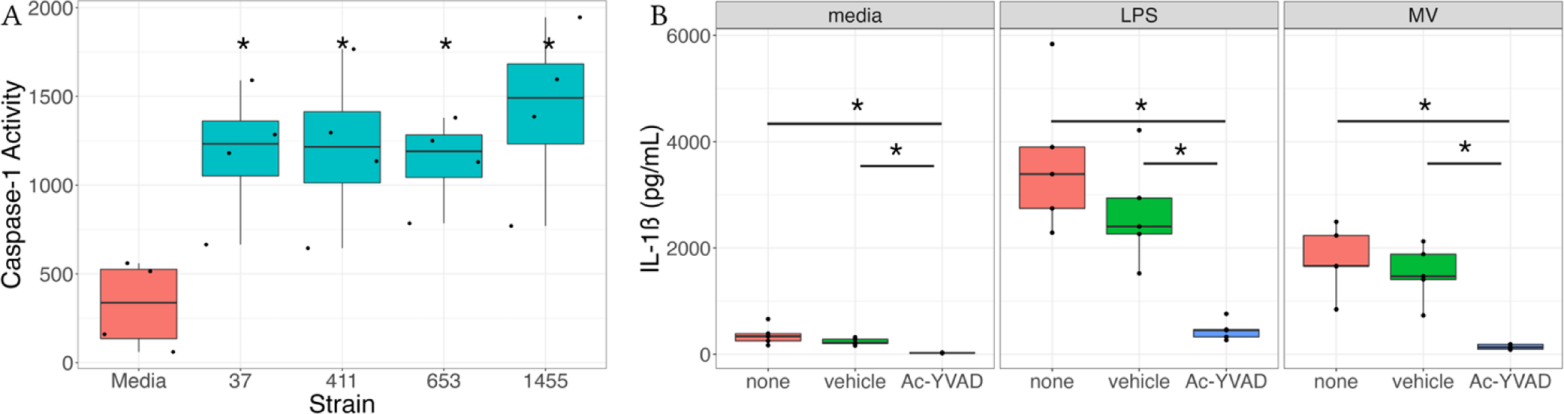
Caspase-1 is critical for the host response to GBS MVs. (A) THP-1-derived macrophages were unstimulated or treated with MVs for 25 h. Supernatants were then assessed for caspase-1 activity using a caspase-1 GLO assay. Relative light units (RLU) were obtained from a GLO Max Navigator. Data represent the amount of caspase-1 activity (Caspase 1 activity = ((RLU GLO reagent) – (RLU GLO Reagent + Ac-YVAD-CHO)) from paired samples. (B) THP-1-derived macrophages were pretreated media, ethanol (vehicle), or Ac-YVAD-CHO for 30 min prior to stimulation with LPS, media, or MVs derived from GB411 for 25 h. Supernatants were then assessed for IL-1β concentration using ProcartaPlex bead-based assays. Individual black dots indicate a single biological replicate (*n* = 4 for each group). Statistical significance is defined as *p* < 0.05 as calculated by ANOVA with a Tukey post hoc and indicated by (*).

Since IL-1β was increased in response to all GBS MVs regardless of the strain, we hypothesized that GBS MVs may induce activation of the inflammasome, a system that is critical for IL-1β secretion and which is known to be induced in response to GBS. Using the Caspase-GLO 1 assay, we detected caspase-1 activity in our untreated controls as well as our LPS-stimulated control, albeit at a substantially higher magnitude in the LPS control (Figure S5). Detectable caspase-1 activity was also observed in response to both the MVs and GBS strains, although some differences were noted. Compared to untreated controls, MVs from the three invasive strains (GB37, GB411, and GB1455) induced the most potent caspase-1 responses, confirming that MVs induce caspase-1 activity (Figures 3A and S5). Among these strains, GB411 bacteria induced a higher degree of caspase-1 activation compared to untreated controls (Figure S6).

To determine if alternative pathways play a role in the conversion of pro-IL-1β to mature active IL-1β, we pretreated THP-1 cells with the capsase-1 inhibitor Ac-YVAD-CHO for 30 min prior to MV or LPS treatment. Both the LPS and untreated controls produced lower amounts of IL-1β when pretreated with Ac-YVAD-CHO compared to the vehicle controls yielding an 83% and 90% reduction, respectively (Figure 3B). Inhibition of caspase-1 by Ac-YVAD-CHO also resulted in almost complete abrogation (91% reduction) of MV-stimulated IL-1β secretion compared with the vehicle control. Importantly, alterations in IL-1β production were not associated with cell death (Figure S7). These findings demonstrate that caspase-1 activation is necessary for the maturation of pro-IL-1β to mature IL-1β in response to GBS MVs, regardless of strain type (Figure 3B).

### NLRP3 Is Essential for MV-Mediated IL-1β Secretion

**Figure 4:**
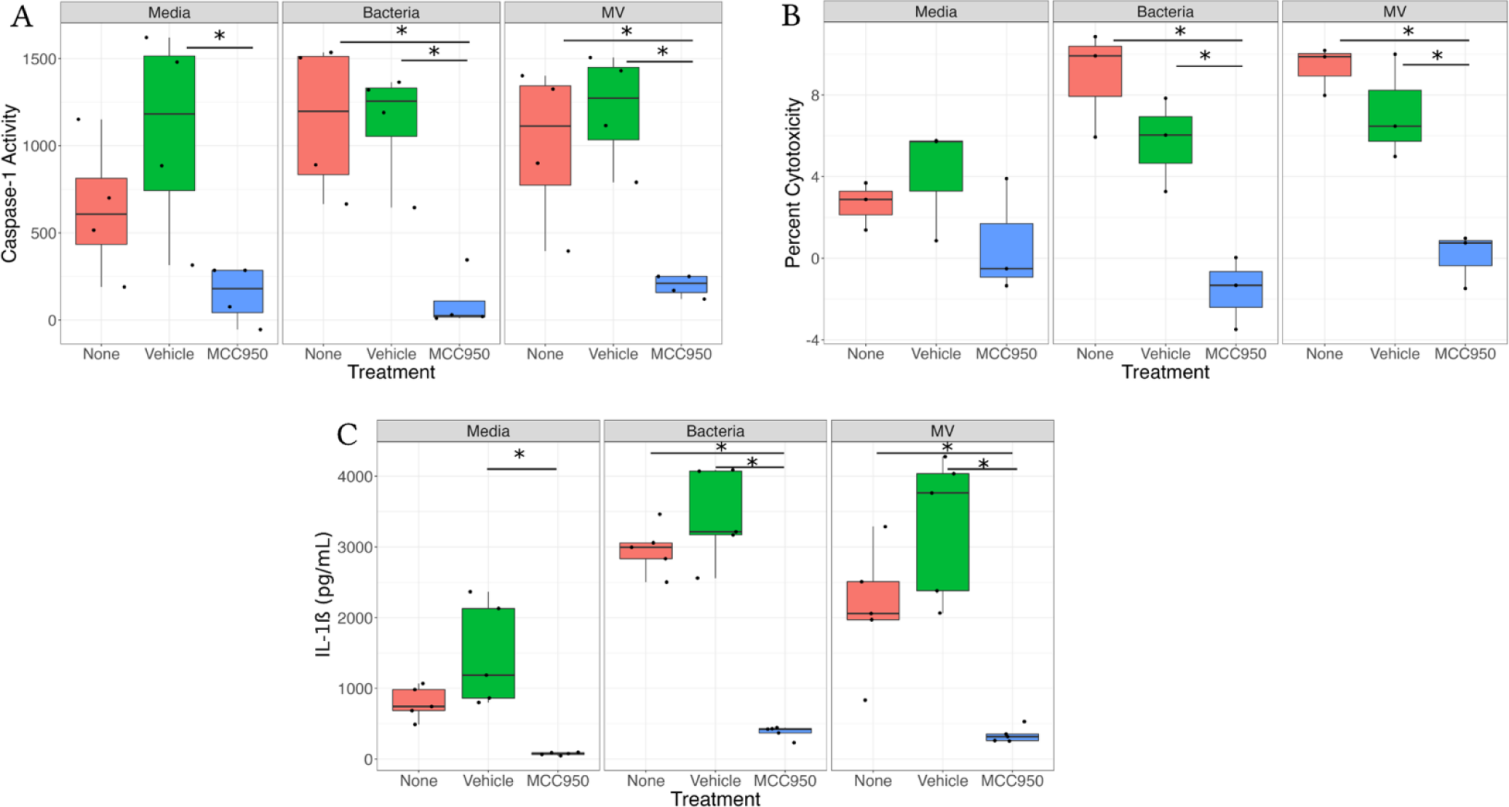
Inhibition of NLRP3 ablates caspase-1 activity in response to MVs. THP-1s were treated with the NLRP3 inhibitor MCC950 or DMSO for 30 min prior to treatment with GB411 bacteria, GB411 MVs, or media for 25 h. (A) Caspase-1 activity was determined using the Caspase-1 GLO assay. Caspase 1 activity = ((RLU GLO reagent) – (RLU Ac-YVAD-CHO + GLO reagent)). Individual points represent individual biological replicates (*n* = 4 each group). (B) Supernatants were assessed for cytotoxicity using the CyQuant LDH Assay. Individual black dots indicate a single biological replicate (*n* = 3 for each group). (C) IL-1β contained in supernatants was quantified using ProcartaPlex IL-1β single plex assays. Individual points represent individual biological replicates (*n* = 5 each group). Statistics were determined using ANOVA with a Tukey’s HSD post hoc test. Significance was defined as *p* < 0.05 and denoted with an (*).

Since caspase-1 was required for IL-1β maturation, we also investigated the upstream MV sensor. Indeed, a prior study showed that GBS triggers inflammasome activation via an NLRP3-dependent mechanism;^31^ hence, we assessed whether inhibition of NLRP3 would impact caspase-1 activation following MV exposure. In these experiments, inhibiting NLRP3 with the MCC950 inhibitor prevented both MV- and GBS-induced caspase-1 activity (Figures 4A and S8). A similar trend was observed in the untreated cells, indicating a baseline level of inflammasome activity in THP-1 macrophages (Figure 4A). NLRP3 inhibition also reduced cytotoxicity in both the GBS- and MV-treated cells, although this result was not observed in the untreated cells (Figure 4B).

Using a similar approach, we also determined that inhibition of NLRP3 signaling significantly decreased IL-1β secretion in both the media and LPS controls relative to the vehicle controls (Figure 4C). Even though the decrease was significant in both groups, the effect was lower for the untreated cells. Moreover, NLRP3 inhibition reduced IL-1β secretion in response to the bacterial strains and their MVs, demonstrating that MV-induced IL-1β requires NLRP3 (Figure 4C).

## Discussion

Previous studies demonstrated that exposure to GBS MVs induces the recruitment of neutrophils and lymphocytes into the gestational membranes^17^ and the neonatal lung;^19^ however, the signals that perpetuate this influx of leukocytes remain unclear. Herein, we have demonstrated that MVs induce expression of proinflammatory cytokines and chemokines in THP-1 cells, which likely contribute to the inflammatory infiltrate observed *in vivo*.^17,19^ Additionally, we found that MVs induce the production of IL-1β by activating pro-IL-1β maturation in an NLRP3- and caspase-1-dependent manner.

By expanding our current understanding of the cytokine responses toward GBS-derived MVs, we have identified the modulators that likely impact some of the adverse pathologies observed during infection. A previous study showed that the murine chemokine KC, known as CXCL1 in humans, was upregulated in response to GBS MVs.^17^ In support of these findings, we found that CXCL1 and many other chemokines are upregulated in THP-1 cells following exposure to GBS MVs. Notably, CCL1, CCL20, CXCL1, and CXCL10 were upregulated in response to MVs from all four clinical strains. The level of chemokine CCL2 was also elevated in response to MVs from the three invasive strains examined. These chemokines are critical for the recruitment of leukocytes to sites of infection with varying target cell specificities. CXCL1 and CCL20, for example, attract neutrophils,^32−34^ whereas CCL1 and CCL2 attract monocytes and macrophages.^35,36^ Additionally, CCL20 and CXCL10 recruit lymphocytes.^34,37^ Unsurprisingly, many of these cytokines have been implicated in GBS-associated disease. CCL20, for instance, was found to be upregulated during infection at the blood–brain barrier,^38^ whereas CCL2 was strongly upregulated in cases with GBS-associated sepsis.^39^ Taken together, these data indicate that GBS MVs serve as a critical initiator of cytokine responses that are commonly linked to disease.

The pyrogen IL-1β, which plays a critical role in the host defense to GBS infection by promoting production of additional neutrophil-specific chemokines,^20,40^ was also upregulated in response to MVs. Although IL-1β lacks direct chemoattractant activity, IL-1β signaling was shown to impact the production of CXCL1 during GBS infection.^20^ In fact, IL1R knockout mice displayed reduced neutrophil recruitment and increased mortality rates when challenged with GBS.^40^ Given the abundant recruitment of neutrophils and lymphocytes into MV-challenged tissues,^17^ these data provide insight into the mechanisms driving this leukocyte infiltration. Although we and others have shown strain variation in IL-1β production in response to bacterial exposure,^13,41^ GBS MVs elicited consistent levels of IL-1β from THP-1 cells, highlighting their potential to serve as important biomarkers or therapeutic targets.

Previous studies have defined the signaling pathways involved in the production of mature IL-1β. For example, high levels of this cytokine were only produced when both toll-like receptor (TLR) signaling and inflammasome activation occurred.^42^ TLR signaling occurs when pathogen-associated molecular patterns (PAMPs) engage their cognate receptor,^43,44^ resulting in the induction of proinflammatory gene expression, including the inactive form of this cytokine, pro-IL-1β.^44^ Canonically, the induction of pro-IL-1β gene expression depends on the translocation of the transcription factor NF-κB into the nucleus.^45^ For pro-IL-1β to be secreted in its mature, active form, a second signal is required, typically in the form of a danger-associated molecular pattern (DAMP), such as a change in membrane potential due to membrane damage,^46,47^ which are sensed by NLRPs.^46,48^ Once sensed, NLRPs oligomerize with other subunits to form the inflammasome,^46,49,50^ which cleaves pro-caspase-1 into its mature, active form.^50,51^ Following inflammasome activation, caspase-1 cleaves pro-IL-1β, thereby triggering its release.^51^ Altogether, this concerted process results in the release of stored pools of pro-IL-1β, allowing for rapid immune activation.

Herein, we demonstrate that GBS MVs triggered caspase-1 activation in THP-1 cells and that the secretion of IL-1β is dependent on caspase-1 activation. We also showed that caspase-1 activation is ablated in the absence of NLRP3 activity, suggesting that NLRP3 is a sensor of GBS MVs. While previous reports have demonstrated that GBS induces IL-1β production in an NLRP3-dependent manner,^28,31,52^ no prior studies have demonstrated that GBS MVs contribute to this response. Because several of the cytokines that were upregulated in response to MVs have not been shown to be regulated by the inflammasome, it is likely that other pattern recognition receptors recognize GBS MVs as well. Nonetheless, we are unaware of additional studies that have identified specific pattern recognition receptors that can sense GBS MVs. That being said, several studies have demonstrated that whole GBS bacteria can be recognized by a plethora of pattern recognition receptors including surface TLRs, such as TLR 2, and endosomal TLRs 7, 8, and 9.^53−56^ Activation of these receptors could prime the production of pro-IL-1β, as well as induce the expression of chemokines, as previously shown.^55,56^ Since our data demonstrate that MVs activate both IL-1β and chemokine production, we hypothesize that priming via TLRs likely occurs in response to MVs. While MVs are likely sensed by surface-level TLRs, a previous study demonstrated that HeLa cells can uptake MVs, indicating that cytosolic TLRs may also be playing a role to facilitate this priming.^17^ This new knowledge may guide the development of receptor antagonist therapies targeting the NLRP3-dependent recognition of GBS MVs, which could prevent host inflammation and subsequent adverse pregnancy outcomes.

We also demonstrated that inhibition of the NLRP3 inflammasome contributed to a reduction in the MV-induced cytotoxicity of THP-1 cells *in vitro*. Moreover, caspase-1 activity was correlated with enhanced cytotoxicity, suggesting that the inflammasome plays a critical role in MV-mediated cytotoxicity. Several studies have shown that some GBS virulence factors, such as hemolysin, can induce NLRP3-dependent pyroptosis^31,52,57,58^ that is mediated by the activation of the pore-forming mediator of pyroptosis, gasdermin D.^59,60^ In THP-1 cells, both the MVs and GBS contributed to a modest amount of cell death, which was dependent on the NLRP3 inflammasome. This finding therefore suggests that MVs may induce pyroptosis through the activation of gasdermin D. Although further studies are needed to confirm this hypothesis, the high levels of IL-1β production and evidence for NLRP3-mediated cell death indicate that MVs may partly contribute to pyroptosis.

A prior study demonstrated that GBS MVs contain active hemolysin and that MV-associated hemolysin exacerbates neonatal sepsis *in vivo*.^19^ Despite the suggestion that GBS-mediated caspase-1 induction requires GBS hemolysin,^31^ our data demonstrate that MVs from the nonhemolytic GB37 strain^61^ still induce a robust IL-1β response and activate caspase-1. Indeed, previously our group has demonstrated that GB37 is capable of inducing IL-1β, NLRP3 activation, and membrane permeabilization in cell lines and primary placental macrophages, suggesting that alternative nonhemolysin mechanisms are capable of inducing this pathway.^62^ This finding indicates that other unknown MV-associated factors also induce caspase-1 activation. Indeed, our prior study, which utilized proteomics to characterize MV-associated proteins, demonstrated that MVs from strains with different genetic backgrounds contain multiple virulence factors that have been linked to inflammatory responses.^22^ Several factors known to promote immune evasion, such as hyaluronidase, sialidase, and C5a peptidase, were detected in GBS MVs at variable levels across strain types.^22^ While these factors can diminish host sensing of GBS, other MV-derived factors likely promote these inflammatory responses. Future studies are therefore required to classify the role that other factors play in activating these signaling cascades, specifically in the nonhemolytic strain GB37.

Despite advancing our current understanding of the host response elicited toward GBS MVs, it is important to recognize the limitations of our study. Although no strain-specific responses toward GBS MVs were observed, increasing the number of isolates may be necessary to detect differences. Furthermore, the cytokine analysis was limited to those included in the antibody microarray; hence, other important responses may have been missed. Although our results are consistent with previous reports regarding the host response to GBS MVs,^17,19^ our system may lack the appropriate complexity to fully model these responses. While THP-1 cells have been shown to largely recapitulate the responses elicited from peripheral blood mononuclear cells, the magnitude of the responses can vary, suggesting that THP-1s can serve as a screening tool for determining cytokine responses from PBMCs.^63^ Previous studies have also demonstrated that neutrophils, which are commonly recruited to the site of GBS infections, produce enzymes capable of cleaving pro-IL-1β.^64^ Consequently, other noninflammasome mechanisms could drive IL-1β maturation *in vivo*, and hence, future studies in alternative model systems with enhanced complexity are warranted.

Dosage of MVs remains a critical limitation of the field. *In vivo* production of MV remains difficult to assess due to the production of host exosomes and vesicles, which are indistinguishable from bacterial MVs via nanoparticle tracking analysis. Previous studies examining the host responses to GBS MVs have utilized a dosage of approximately 10–250 ug/mouse or 30 ug/well of HeLa cells. When normalizing our data to protein concentration, we utilize less than 0.1 ug of MVs/well in our assays (i.e., 100–2500× less by our estimates). Together we believe that these data suggest that we are using a more physiologic dosage of GBS or GBS MVs than other studies in the field.^17,19^ That being said, future studies aimed at determining the physiologic production of MVs *in vivo* are of critical importance.

Another critical limitation of this study is that tetrapeptide inhibitors, such as MCC950, often have some low level of nonspecific binding, which could limit our interpretation of these data. Previous studies using knockouts of NLRP3 have demonstrated that IL-1β responses to GBS are primarily driven by the NLRP3-inflammasome rather than noncanonical inflammasomes or receptors, suggesting that off-target effects are unlikely.^31^ Furthermore, in the assays used in this study, very little nonspecific activity has been observed, suggesting that off-target effects are unlikely.^65^ That being said, future studies using mouse models and genetic knockouts may be necessary to confirm these results.

**Figure 5:**
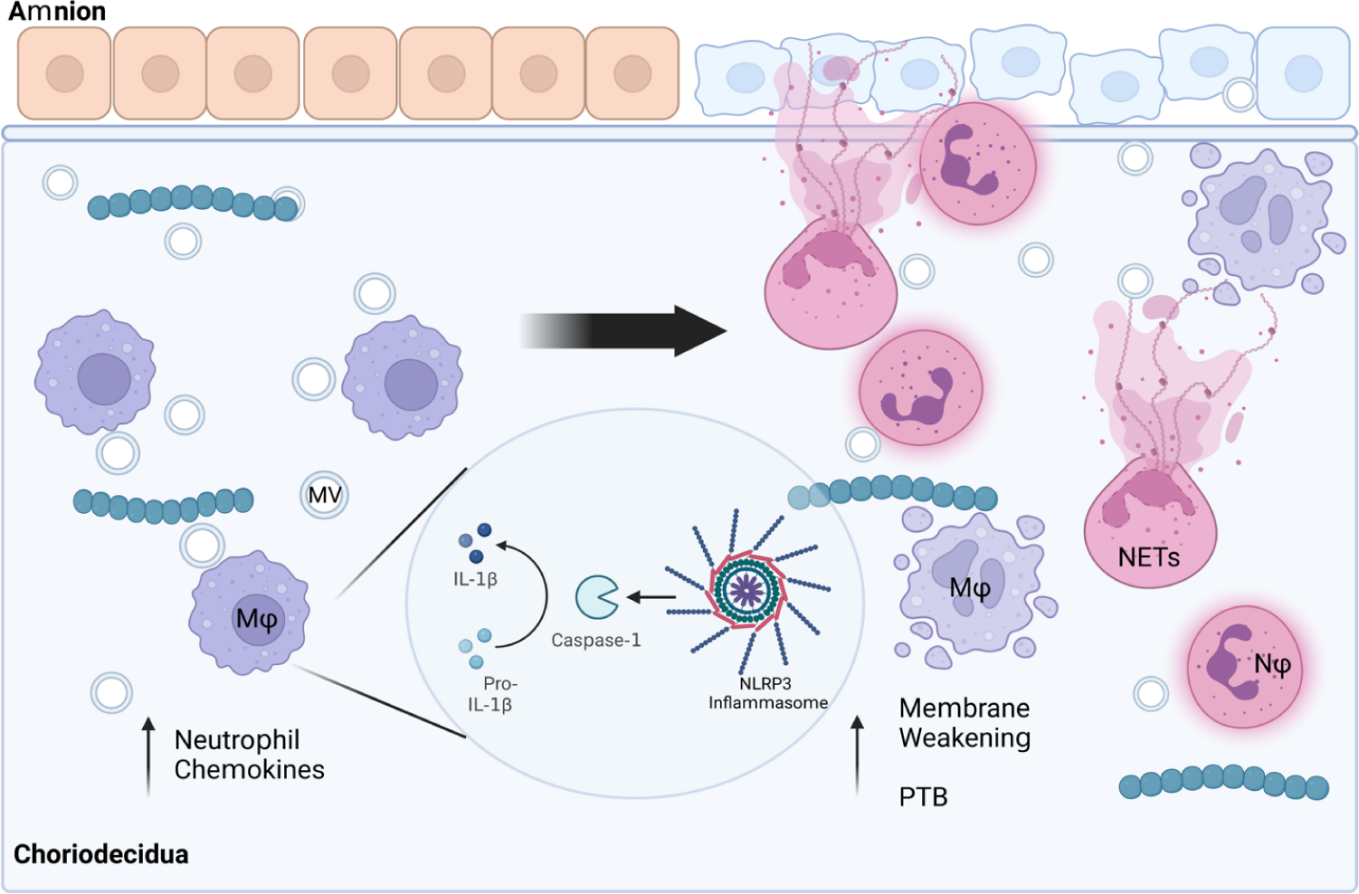
Model of GBS-mediated chorioamnionitis. GBS is a frequent cause of chorioamnionitis. As sentinel cells at the maternal–fetal interface, macrophages play a critical role in shaping how inflammatory responses are initiated. We show here that macrophages respond to MVs by releasing proinflammatory cytokines and chemokines, many of which recruit neutrophils to the site of infection. Additionally, we show that MVs activate the NLRP3 inflammasome, triggering release of the pyrogen IL-1β. Together these processes promote an influx of neutrophils and leukocytes into the site of infection. In cases such as chorioamnionitis, neutrophils undergo processes including NET-osis, which promote tissue weakening and subsequent preterm birth. Taken together, these findings demonstrate mechanistically how MVs may promote preterm birth and chorioamnionitis *in vivo*.

Regardless of these limitations, data from this study enhance our understanding of how GBS MVs promote both adverse pregnancy and neonatal infection outcomes (Figure 5). It has been established that GBS MVs promote adverse outcomes partly by enhancing neutrophil recruitment.^17,19^ In conditions such as chorioamnionitis, we suggest that the sensing of MVs by macrophages may promote proinflammatory immune signaling. Consistent with these findings, we have demonstrated that MVs promote the release of many neutrophil-recruiting chemokines as well as the pyrogen IL-1β, which are important for neutrophil recruitment that promote tissue damage via NETosis.^20,34,66,67^ We also demonstrate that the MV-mediated induction of IL-1β is dependent on caspase-1 activation, which further promotes a proinflammatory environment. Through both direct and indirect tissue damage, MVs likely play a role in weakening gestational membranes, inducing chorioamnionitis, and promoting preterm labor due to enhanced induction of these inflammatory responses (Figure 5). Collectively, these findings expand our understanding of how the immune system responds to these bacterial components that contain important virulence factors capable of initiating an inflammatory response. While the specific PAMPs and DAMPs contained in MVs are not known, this study provides a foundation for future studies aiming to classify the specific factors within MVs that trigger these responses.

Together, these data illustrate that GBS MVs can induce potent proinflammatory cytokine responses in human-derived THP-1 cells, which are due in part to the activation of the NLRP3 inflammasome. This study advances our understanding of how GBS MVs interact with the host by classifying the cytokine response toward GBS MVs and through the identification of NLRP3 as a sensor of MVs. Since these cytokine responses are largely conserved across genetically distinct strains, they could serve as targets for immunotherapy or biomarkers to rapidly determine disease status in future studies. This study has enhanced our mechanistic understanding of how GBS MVs promote inflammatory conditions, such as preterm labor, and serves as a critical baseline for future studies aimed at examining the host response to GBS MVs *in* vivo using animal models, or *in* vitro using primary cell culture.

## Conclusions

Previous reports have demonstrated that GBS MVs are potent virulence factors that promote inflammatory infiltration during preterm birth and neonatal sepsis, although the specific immune effectors responsible for this response are not clear. Herein, we demonstrated that GBS MVs induced specific and conserved cytokines and chemokines from THP-1 cells, including the pyrogen IL-1β. We further demonstrated that IL-1β production is dependent on the activation of NLRP3-inflammasome, a novel sensor of GBS MVs. Collectively, these data expand our understanding of how the host senses and responds to GBS MVs and have identified potential immunotherapeutic targets for the prevention of GBS-induced disease.

## Methods

### Bacterial Strains and Culture

GBS strains GB00037 (GB37), GB00411 (GB411), GB00653 (GB653), and GB01455 were isolated as described previously.^68,69^ The invasive isolates GB37, GB411, and GB1455 were isolated from the blood or cerebrospinal fluid of infants with early-onset GBS disease,^68^ while the colonizing strain (GB653) was isolated from a vaginal/rectal swab of an asymptomatic mother before childbirth.^69^ These isolates were previously characterized by MLST and capsular serotyping^9,11^ and were found to represent three common STs, namely, ST-1 (GB37, serotype V), ST-12 (GB1455, serotype II and GB653, serotype II), and ST-17 (GB411, serotype III). Further, previous characterization of GB37 demonstrated that GB37 is nonhemolytic while retaining its invasive phenotype.^61^ Strains were cultured using Todd-Hewitt Broth (THB) or Todd-Hewitt Agar (THA) (BD Diagnostics, Franklin Lakes, New Jersey, USA) overnight at 37 °C with 5% CO_2_.

### Membrane Vesicle (MV) Isolation

Previous studies examining GBS MVs have relied solely on ultracentrifugation for the isolation of MVs. To improve the purity of MVs, we used ultracentrifugation coupled with size exclusion chromatography to remove extravesicular contaminants, as we have described previously.^22^ Briefly, overnight THB cultures were diluted 1:50 into fresh broth and grown to the late logarithmic phase (optical density (OD)_600_ = 0.9). Cultures were centrifuged at 2000*g* for 20 min at 4 °C. Supernatants were collected and recentrifuged at 8500*g* for 15 min at 4 °C, followed by filtration through a 0.22 μm filter and concentration using Amicon Ultra-15 centrifugal filters (10 kDa cutoff) (MilliporeSigma, Burlington, MA, USA). Concentrated supernatants were subjected to ultracentrifugation for 2 h at 150,000*g* at 4 °C. Pellets were resuspended in PBS and purified using qEV single size exclusion columns (IZON Science, Christchurch, New Zealand) per the manufacturer’s instructions. MV fractions were collected and reconcentrated using the Amicon Ultra-4 centrifugal filters (10 kDa cutoff) (MilliporeSigma, Burlington, Massachusetts, USA) to a final volume of 100 μL in PBS. MVs were aliquoted and stored at −80 °C until further use.

### Nanoparticle Tracking Analysis

MVs were quantified via nanoparticle tracking analysis using a NanoSight NS300 (Malvern Panalytical Westborough, MA, USA) equipped with an automated syringe sampler as described previously.^22,70,71^ For each sample, MVs were diluted in PBS (1:100–1:1000) and injected at a flow rate of 50. Once loaded, five 20 s videos were recorded at a screen gain of 1 and camera level of 13, which were analyzed at a screen gain of 10 and a detection threshold of 4 after capture. Data were subsequently exported to a CSV file for analysis using the R package tidyNano.^70^

### THP-1 Cell Culture

THP-1 cells (TIB-202) were obtained through ATCC (Manassas, VA) and stored according to vendor guidelines.^72^ The cells were cultured in RPMI 1640 (Gibco, Thermo Fisher, Waltham, MA) supplemented with l-glutamine, 10% fetal bovine serum (FBS), and a 1% antibiotic-antimycotic solution (100 μg/mL streptomycin, 0.25 μg/mL amphotericin B, and 100 U/mL penicillin; Gibco, Thermo Fisher, Waltham, MA) as described.^12,13^ For experiments, THP-1 cells were utilized only until passage 10. When indicated, THP-1 monocytes were differentiated into macrophages using phorbol 12-myristate 13-acetate (PMA) as previously described.^12,13^ Cells were differentiated in RPMI (without phenol red) supplemented with l-glutamine, 2% FBS, and 100 nM PMA for 24 h prior to experimentation.^12,13^

For experiments using GBS-treated cells, THP-1 cells were washed twice with PBS prior to infection. The bacteria were resuspended in RPMI and added to the THP-1 cells at a multiplicity of infection (MOI) of 10 bacteria per cell. Cells were incubated for 1 h, and the media was aspirated. Cells were washed thrice with PBS, and fresh RPMI with l-glutamine (no phenol red) containing 2% FBS, 100 nM PMA, penicillin (5 μg/mL), and gentamicin (100 μg/mL) was added (termed RPMI 2/0). Cells were incubated for an additional 24 h. For MV treatments, cells were washed twice, and fresh RPMI 2/0 containing MVs at an MOI of 100 MVs per differentiated macrophage was added and incubated for 25 h. Cells were treated with lipopolysaccharide (LPS) (1 μg/mL, clone L2654, Millipore Sigma, Burlington, MA), which served as a positive control. At the end of each treatment period, supernatants were collected, centrifuged at 4 °C for 10 min at 4000 rev/min, and aliquoted. Samples were stored at −80 °C.

### Cytokine and Cytotoxicity Analysis

Semiquantitative analysis of cytokines was performed on the supernatants from THP-1 cultures using a human cytokine antibody microarray (ab133998, Abcam, Cambridge, UK) as described.^13^ Cells were seeded into six-well plates at a density of 4 × 10^6^ per well and treated with bacteria or MVs at an MOI of 10 and 100, respectively. Membranes were imaged with an Amersham Imager 600 (GE Life Sciences), and densitometry was performed using ImageJ software. Cytokines with more than a 2-fold change relative to mock-treated were considered upregulated and validated quantitatively using ProcartaPlex bead-based assays (Thermo Fisher, Waltham, MA). Cells were seeded into 12-well plates at a density of 2 × 10^6^ cells per well and treated with bacteria or MVs as described above. These assays were performed per manufacturer’s instructions and subsequently read and analyzed using a Luminex 200 and Luminex xPONENT v3.1 software, respectively (Luminex Corp., Austin, Texas). Using the same treatment scheme, cellular cytotoxicity was assessed using a CyQuant lactate dehydrogenase (LDH) assay (Invitrogen, Waltham, MA).

### Caspase-1 Activity, Responses, and Inhibition

After treatment of THP-1 cells, caspase-1 activity was quantified in supernatants using a commercially available assay (Caspase-GLO 1 Assay; Promega, Madison, WI), which was previously shown to be highly sensitive and specific for caspase-1.^65^ Caspase-GLO 1 assays were quantified using a GloMax Navigator instrument (Promega, Madison, WI). To determine the impact of caspase-1 on MV-induced IL-1β production, PMA-differentiated THP-1 cells were seeded into 12-well plates and pretreated with 50 μM caspase-1 inhibitor Ac-YVAD-CHO (Cayman Chemical Company, Ann Arbor, MI), and alternatively 10 μM of the NLRP3-specific inhibitor, MCC950 (Invitrogen, Waltham, MA), for 30 min, which has previously been shown to be selective for the NLRP3 inflammasome.^73^ Cells were treated with either LPS or GBS as described above, and IL-1β concentrations were measured with the ProcartaPlex simplex assay (Thermo Fisher, Waltham, MA).

### Data Analysis

Data analysis was performed using RStudio.^74^ Shapiro–Wilk tests were used to determine whether the data followed a normal distribution. Normally distributed data were analyzed for significance using a two-way analysis of variance (ANOVA), followed by a Tukey HSD *post hoc* test. By contrast, nonparametric data were analyzed using a Kruskal–Wallis test, followed by Dunn’s *post hoc* test to detect differences between groups. Multiple hypothesis testing was corrected using a Benjamini–Hochberg or Bonferroni correction when necessary. The analyses used for individual experiments are denoted in the figure legends for the sake of clarity.

## Supporting Information Available

Cytokine antibody microarray data (Figure S1); luminex data assessing cytokine responses to whole bacteria (Figure S2); luminex data for IL-6 responses to MVs and bacteria (Figure S3);LDH assay in response to MVs and bacteria (Figure S4);unnormalized caspase GLO assay data (Figure S5); normalized caspase GLO results for bacterial controls (Figure S6); LDH assays during caspase-1 inhibition (Figure S7); and NLRP3 inhibition and its impact on caspase-1 activity (Figure S8) (PDF)
