# Supplementary material for: Group B Streptococcal Membrane Vesicles Induce Proinflammatory Cytokine Production and Are Sensed in an NLRP3 Inflammasome-Dependent Mechanism in a Human Macrophage-like Cell Line
Source: ACS Infect Dis. 2025 Jan 6;11(2):453–62. doi: 10.1021/acsinfecdis.4c00641 (PMC11833861; doi:10.1021/acsinfecdis.4c00641)
Supplement: Supplementary file 1 — id4c00641_si_001.pdf [file id4c00641_si_001.pdf]

Supporting Information

Group B streptococcal membrane vesicles induce proinflammatory cytokine production and are sensed in an NLRP3 inflammasome-dependent mechanism in human macrophage-like cell line

Cole R. McCutcheon [1], Jennifer A. Gaddy [2,3,4], David M. Aronoff [5], Shannon D. Manning [1*], and Margaret G. Petroff [1,6*]

[1] Department of Microbiology, Genetics, and Immunology, Michigan State University, East Lansing, MI 48824, United States
[2] Department of Medicine, Division of Infectious Disease, Vanderbilt University Medical Center, Nashville, TN 37232, United States
[3] Department of Pathology, Microbiology, and Immunology, Vanderbilt University Medical Center, Nashville, TN 37232, United States
[4] Tennessee Valley Healthcare System, Department of Veterans Affairs, Nashville, TN 37212, United States
[5] Department of Medicine, Indiana University School of Medicine, Indianapolis, IN 46202, United States
[6] Department of Pathobiology and Diagnostic Investigation, Michigan State University, East Lansing, MI 48824, United States

*Address correspondence:
Margaret Petroff, PhD
766 Service Road, Room 3009
East Lansing, MI 48824
petrof10@msu.edu

Shannon Manning, PhD
194 Food Safety & Toxicology,
East Lansing, MI 48824
mannin71@msu.edu

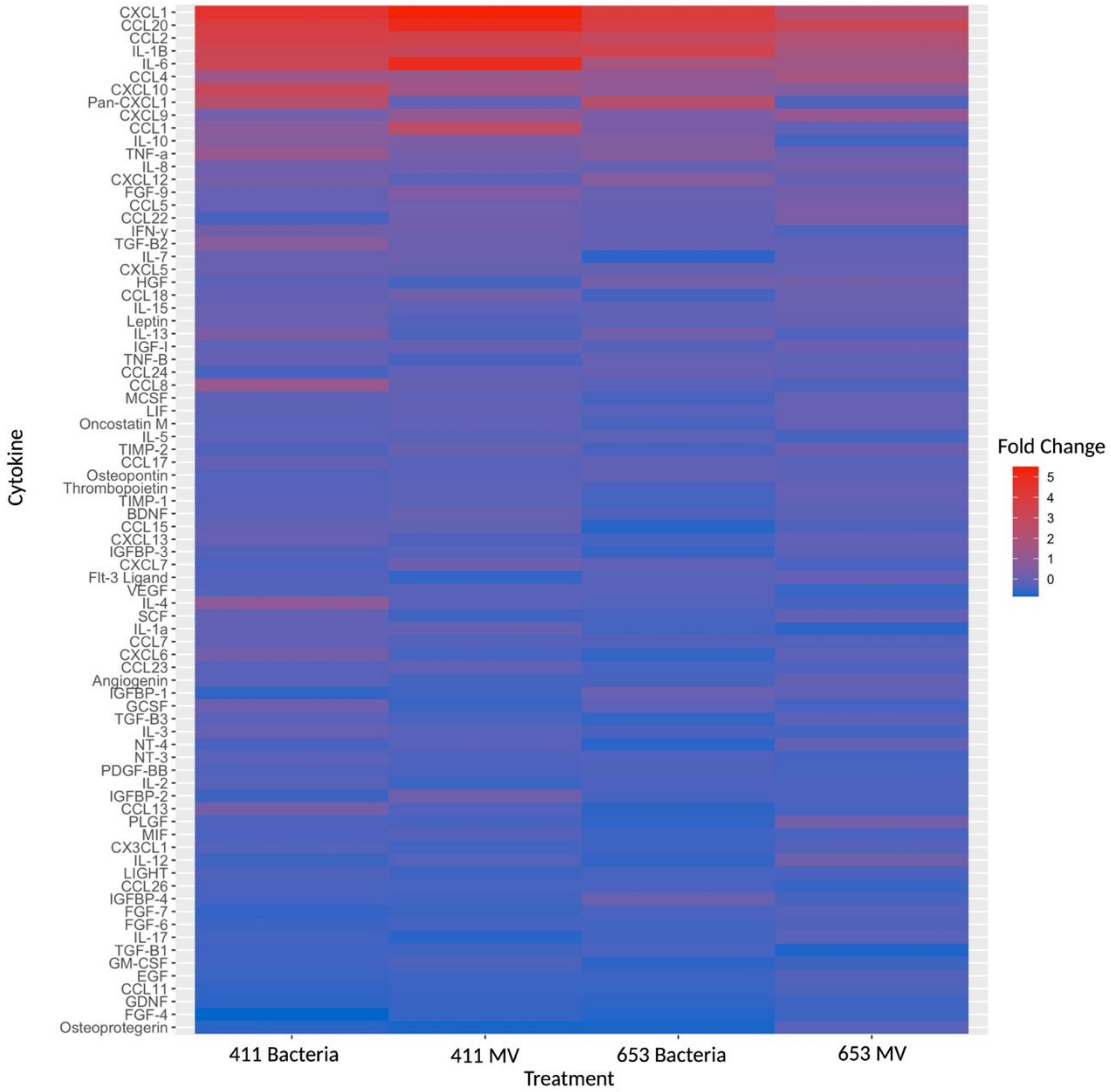

Figure S1: Profiling of cytokine responses elicited towards MVs

THP-1s were treated with bacteria (multiplicity of infection (MOI) = 10) or MVs (MOI 100) for 25 hours prior to supernatant collection. Cytokine production was analyzed using a human cytokine antibody microarray (Abcam). Shown here is semi-quantitative densitometry analysis (ImageJ) of cytokine production for all 80 cytokines examined. Color denotes fold change relative to untreated controls. All groups were performed in biological duplicate. Boxes indicate mean fold change for each condition.

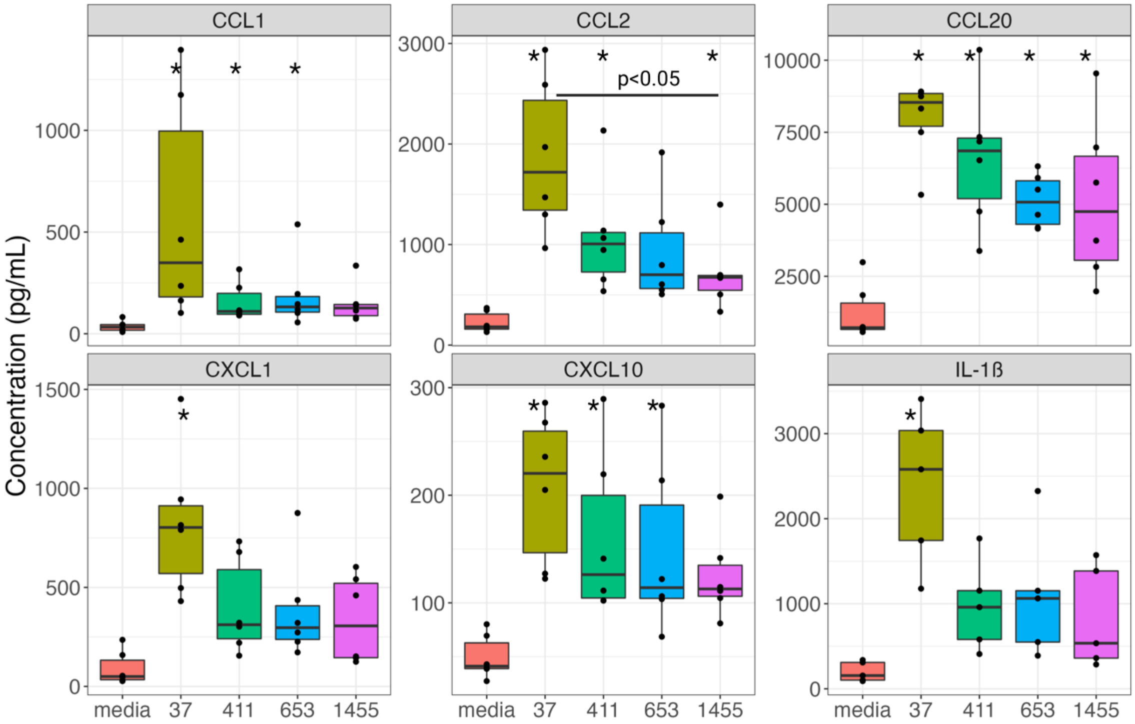

Figure S2: Bacteria elicit proinflammatory immune responses from THP-1 macrophages. Supernatants from THP-1 derived macrophages, which were untreated or treated with bacteria (MOI 10) for 25 hours were assessed for cytokine production using ProcartaPlex multiplex bead-based assays. Each black dot indicates a single biological replicate (n = 5-6 for each group). Data were analyzed by one-way ANOVA with a Tukey HSD post hoc test, or for non-parametric data, a Kruskal Wallis test with a Dunn Test post hoc test. Comparisons with $p < 0.05$ relative to untreated are denoted with (*). Significant differences between strains are denoted with a specific p-value.

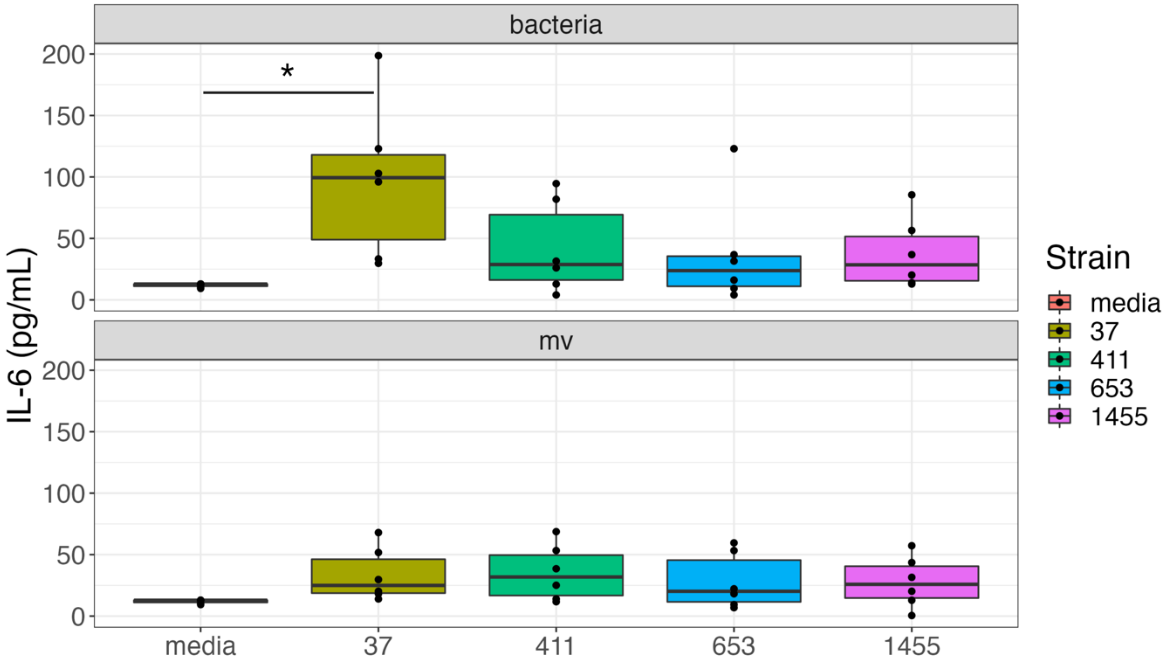

Figure S3: IL-6 is not produced in response to GBS MVs

Supernatants from unstimulated or MV-treated THP-1 derived macrophages were assessed for IL-6 using ProcartaPlex multiplex bead-based assays. Individual black dots indicate a single biological replicate (n = 5-6 for each group). Statistics were determined by one-way ANOVA with a Tukey HSD post hoc, or for non-parametric data, a Kruskal Wallis test with a Dunn post hoc test. Significantly different comparison between groups (P-value < 0.05) are denoted with (*).

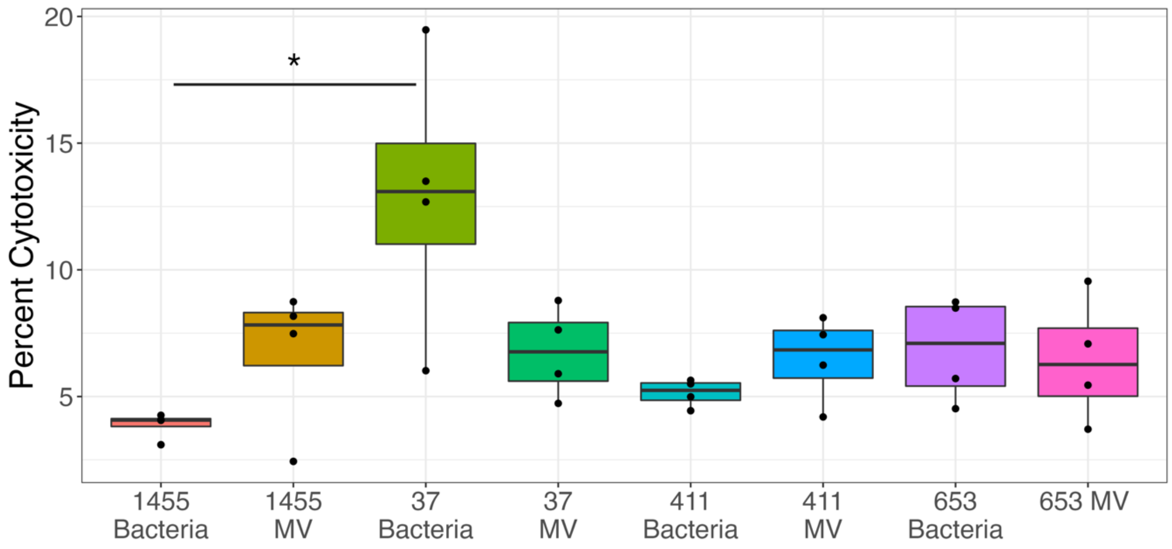

Figure S4: MVs induce a low amount of cell death in THP-1s

THP-1 derived macrophages were unstimulated or treated with MVs for 25 hours. Supernatants were assessed for cytotoxicity using the CyQuant LDH Assay. Percent Cytotoxicity is expressed as a percentage relative to untreated cells. Each black dot represents a single biological replicate (n = 4 /group). Data were analyzed using either a one-way ANOVA with a Tukey HSD post hoc test (MV treated groups), or a Kruskal Wallis test with a Dunn Test post hoc (Bacteria-treated groups). Significantly different comparison within groups (P-value < 0.05) are denoted with (*). All other comparisons were not significantly different.

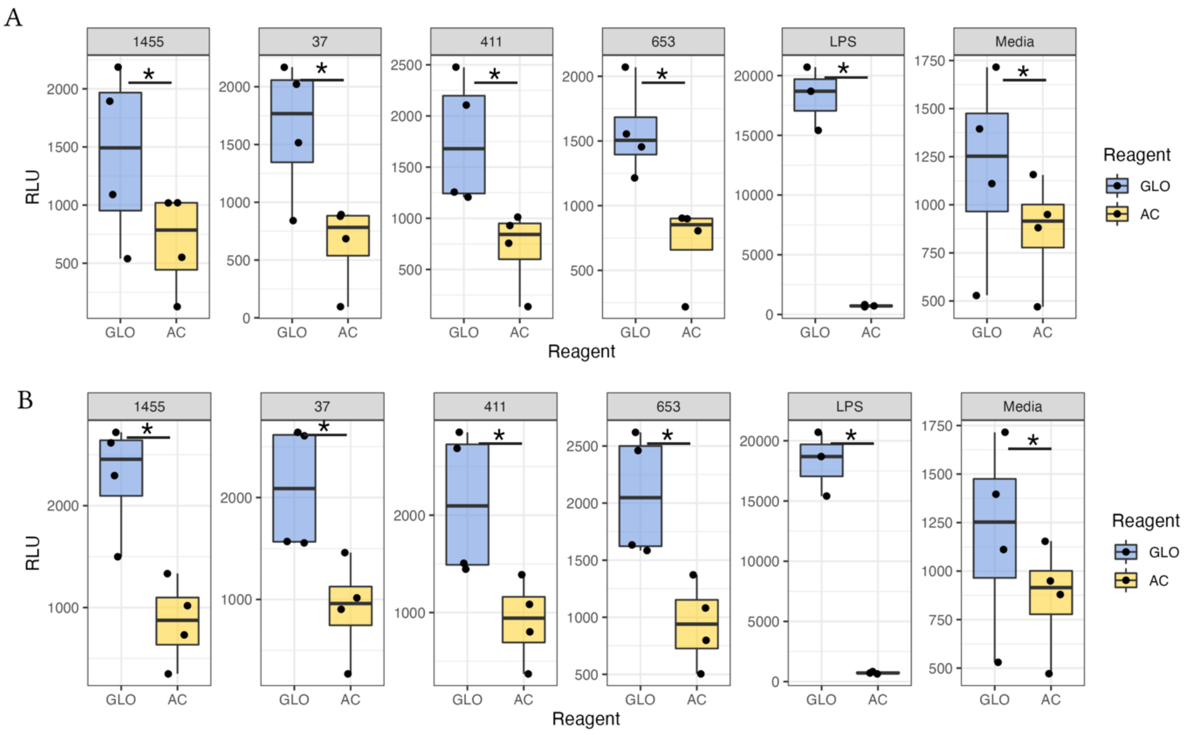

Figure S5: MVs induce caspase-1 activity
Supernatants from THP-1 derived macrophages which were unstimulated or treated with bacteria, MVs for 25 hours. Alternatively, cells were stimulated with LPS for 2 hours. Supernatants were then assessed for caspase-1 activity using a caspase-1 GLO assay. GLO refers for Caspase GLO assay activity, whereas AC refers to Caspase GLO activity including the caspase-1 inhibitor Ac-YVAD-CHO. A.) Activity from THP-1s treated with bacteria. B.) Activity from THP-1s treated with MVs. Relative light units (RLU) were determined using a GLO Max Navigator. Individual black dots indicate a single biological replicate (n = 3-4 for each group). Statistics were determined using a two-sided, paired t-test. P-value < 0.05 relative to mock treatment is denoted with a (*).

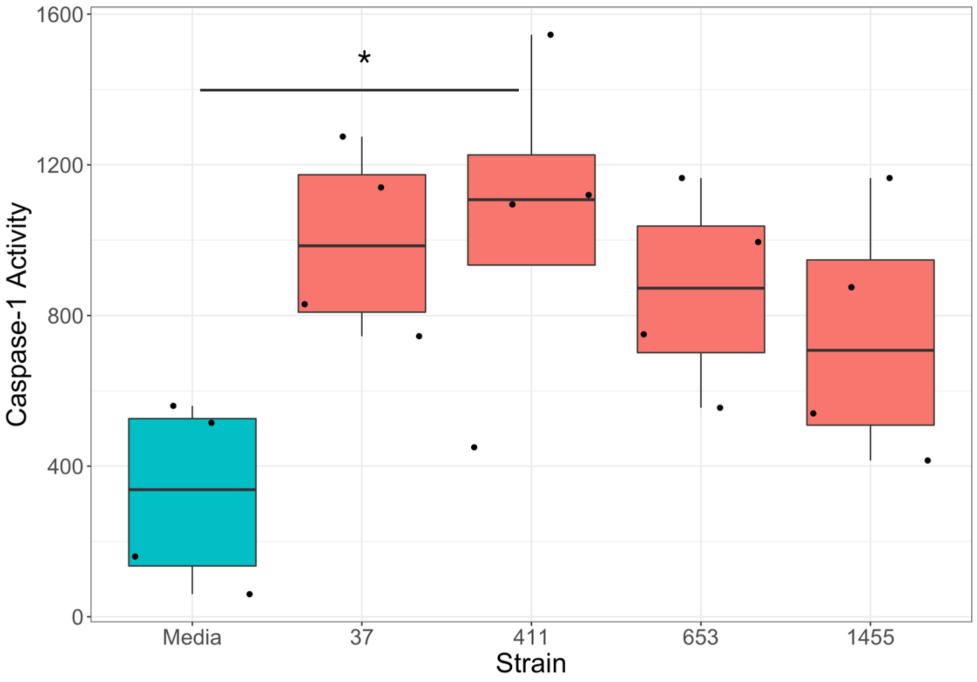

Figure S6: Bacteria induce caspase-1 activation in THP-1s

THP-1 derived macrophages were unstimulated or treated with bacteria for 25 hours. Supernatants were then assessed for caspase-1 activity using a caspase-1 GLO assay. Data represent the amount of caspase-1 activity (Caspase 1 activity = ((RLU GLO reagent) – (RLU Ac-YVAD-CHO + GLO Reagent)) from paired samples. Individual black dots indicate a single biological replicate (n = 4 for each group). Statistical significance is defined as $p<0.05$ as calculated by ANOVA with a Tukey post-hoc and indicated by (*).

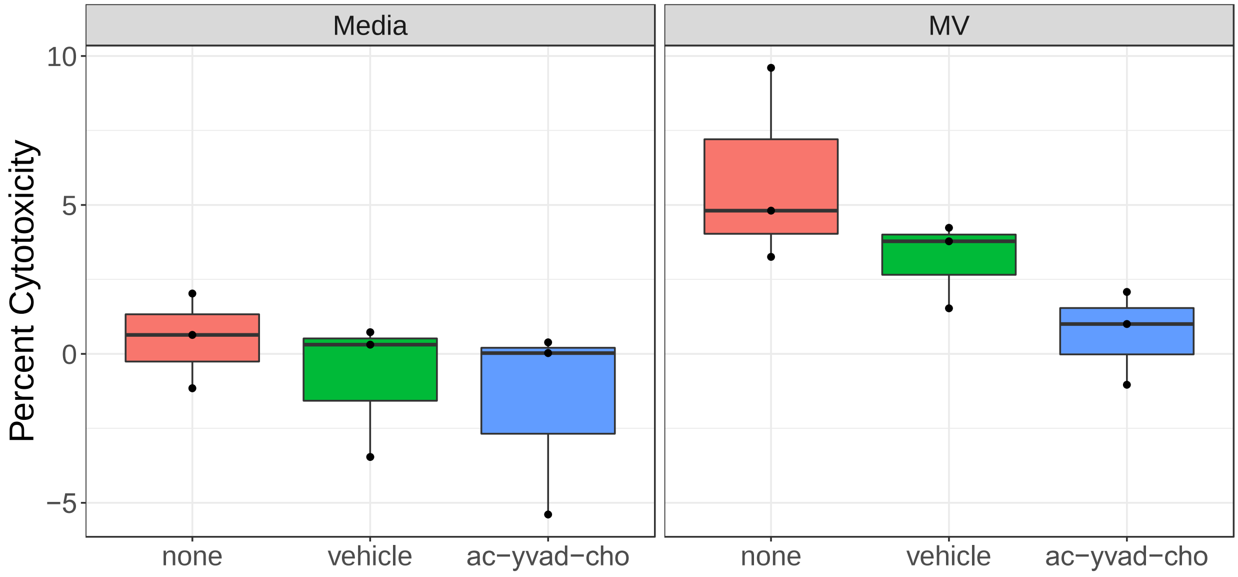

Figure S7: Caspase-1 inhibition does not impact cell death responses

THP-1s were untreated, treated with ethanol, or Ac-YVAD-CHO for 30 minutes. Supernatants from THP-1 derived macrophages, which were subsequently unstimulated or treated with GB411 MVs for 25 hours were assessed for cytotoxicity using the CyQuant LDH Assay. Individual black dots indicate a single biological replicate (n = 3 for each group). Statistics were determined using either an ANOVA with a Tukey HSD post hoc. No significant difference relative to non-pretreated cells were detected for either group.

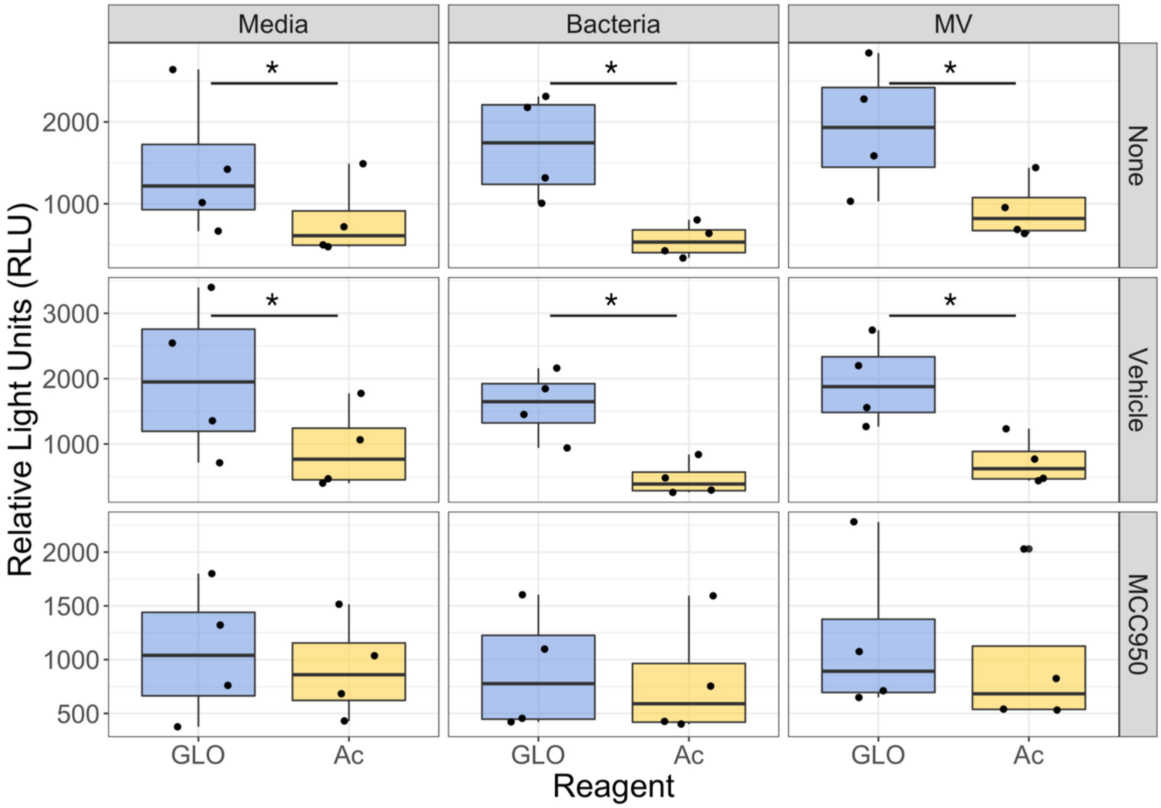

Figure S8: Inhibition of NLRP3 prevents Caspase-1 Activation in Response to MVs

THP-1s were treated with the NLRP3 inhibitor MCC950 prior to treatment with GB411 bacteria, GB411 MVs, or media. Caspase-1 activity was determined using the Caspase-1 GLO assay. GLO refers for Caspase GLO assay activity, whereas AC refers to Caspase GLO activity including the caspase-1 inhibitor Ac-YVAD-CHO. Individual points represent individual biological replicates (n = 4 each group). Statistics were determined using an ANOVA with a Tukey's HSD post-hoc test. Significance was defined as p>0.05 and denoted with an (*).
